# Abnormal Functional Connectivity of Ventral Anterior Insula in Asthmatic Patients with Depression

**DOI:** 10.1155/2017/7838035

**Published:** 2017-06-07

**Authors:** Yuqun Zhang, Yuan Yang, Rongrong Bian, Yingying Yin, Zhenghua Hou, Yingying Yue, Zhi Xu, Yonggui Yuan

**Affiliations:** ^1^Department of Psychosomatics and Psychiatry, Zhongda Hospital, School of Medicine, Southeast University, Nanjing, China; ^2^Institute of Psychosomatics, School of Medicine, Southeast University, Nanjing, China; ^3^Department of Respiration, Zhongda Hospital, Southeast University, Nanjing, China

## Abstract

**Objective:**

To explore the underlying mechanism of depression in asthmatic patients, the ReHo in the insula and its FC was used to probe the differences between depressed asthmatic (DA) and nondepressed asthmatic (NDA) patients.

**Methods:**

18 DA patients, 24 NDA patients, and 60 healthy controls (HCs) received resting-state fMRI scan, severity of depression, and asthma control assessment.

**Results:**

DA patients showed increased FC between the left ventral anterior insula (vAI) and the left middle temporal gyrus compared with both NDA and HC groups. In addition, compared with HCs, the DA and NDA patients both exhibited increased FC between the left vAI and the right anterior cingulate cortex (ACC), decreased FC between the left vAI and the bilateral parietal lobe, and increased FC between the right vAI and the left putamen and the right caudate, respectively. Furthermore, the increased FC between the left vAI and the right ACC could differentiate HCs from both DA and NDA patients, and the increased FC between the right vAI and both the left putamen and the right caudate could separate NDA patients from HCs.

**Conclusions:**

This study confirmed that abnormal vAI FC may be involved in the neuropathology of depression in asthma. The increased FC between the left vAI and the left MTG could distinguish DA from the NDA and HC groups.

## 1. Introduction

Bronchial asthma is a common chronic inflammatory condition that swelled and narrowed the airways, leading to dyspnea, coughing, and tightening of the chest. Asthma is significantly associated with psychiatric disorders [1], especially depression that has been consistently reported to be prevalent in asthmatic patients [2–4]. Adeyeye et al. [5] indicated that depression is the most important factor independently associated with asthma-related quality of life. And an epidemiology study found asthma per se may be an independent risk factor for suicidality [6]. Inflammation-associated mood deterioration was reflected in changes in brain function during evoked responses to emotional stimuli [7].

The development of brain functional magnetic response imaging (fMRI) has been a useful technique to explore the neurobiological mechanisms of asthma and emotion. Rosenkranz et al. [8–10] detected the neural circuitry underlying the interaction between emotion and asthma symptoms used task fMRI, and the findings consistently indicated that neuron phenotypes of asthma might be identified by neural activity of brain circuits previously implicated in emotion regulation, especially the insula.

The insula may modulate inflammatory processes by the influence on neuroendocrine responses to stress, including extensively studied effects on the HPA axis and its physiological responses [11]. As a “cortical hub,” the insula carries information of dyspnea and has strong connections with neural structures important in processing emotional information [8, 12–14]. However, to our knowledge, only one research explored the underpinnings of depression with the method of fMRI in female asthmatic patients, in which depressed asthma (DA) patients showed a decreased spontaneous activity in the right insula [15]. Furthermore, the functional connectivity (FC) between the insula and other brain regions was not clear. Therefore, we selected the insula as a region of interest (ROI) to explore whether its regional homogeneity (ReHo) and FC changes occur with depression in asthma. We hypothesized that DA and nondepressed asthma (NDA) patients would show abnormal spontaneous activity in insula and abnormal insula FC compared with that of healthy controls (HCs).

## 2. Materials and Methods

### 2.1. Participants

After attrition and data screening, the sample included 42 patients with a diagnosis of bronchial asthma with nonacute attacks and 60 HCs. 18 of the included asthmatic patients (12 patients with steroid treatment) entered into the DA group and the other 24 patients (18 patients with steroid treatment) entered into the NDA group according to the scores of the 17-item Hamilton Depression Rating Scale (HDRS-17). There was no statistical difference between the DA and NDA patients (*χ*^2^ = 0.350, *P* = 0.554). All participants signed a written informed consent form, as required by the ethics committee (Zhongda Hospital, Southeast University, Nanjing, People's Republic of China). The clinical trial registration number was ChiCTR-COC-15007442.

### 2.2. Inclusion/Exclusion Criteria

Participants were all at least 18 years old, right-handed, and had an educational level of junior high school or higher. Asthmatic patients were diagnosed as bronchial asthma with nonacute attacks. The HCs were required to have a score below 7 on the HDRS-17.

Participants were excluded if they are presented with other serious physical diseases, psychotic disorders, and alcohol or drug dependence; were pregnant or lactating; and had electronic or other metal equipment that was surgically implanted (such as a cardiac pacemaker, defibrillator, and stent).

### 2.3. Evaluations

#### 2.3.1. HDRS-17

In the current study, all subjects received HDRS-17 evaluation by researchers. HDRS-17 [16] contains 17 variables which are measured on five-point scales, and it is used to assess the depression severity. Participants with a score equal or above 7 are recognized having depression possibly.

#### 2.3.2. Asthma Control Test (ACT)

All asthmatic patients completed ACT by themselves. ACT [17] contains 5 items with a total score arranging from 0 to 25. Patients with a total score below 20 are thought to have no control for their asthma.

### 2.4. Brain Image Acquisition

Imaging was performed on a 3-Tesla Scanner using a homogeneous birdcage head coil. Participants were required to keep their eyes closed, awake, and not think of specific things during scanning. Participants lay supine with the head snugly fixed by a belt and foam pads to minimize head motion. A gradient-recalled echo-planar imaging (GRE-EPI) pulse sequence was set up to acquire resting-state images. For each data volume, we acquired 36 continuous axial slices in descending order with 3.75 mm × 3.75 mm in-plane resolution parallel to the anterior commissure-posterior commissure line, 3 mm slice thickness, and a 0 mm gap using resting-state imaging (TR = 2000 ms, TE = 25 ms, flip angle = 90°, acquisition matrix = 64 × 64, field of view = 240 mm × 240 mm). This acquisition sequence generated 240 volumes in 8 minutes.

### 2.5. Functional Imaging Preprocessing

All the image data were reconstructed and inspected by two experienced radiologists. Image preprocessing was performed using the DPARSF software [18]. The first 10 time points were discarded for scanner calibration and for subjects to get used to the circumstance. The remaining time points were corrected for timing differences between slices and for motion effects (six-parameter rigid body) using a reference volume in the center of the run. After head motion correction, participants with head motion of more than 2.5 mm of maximum displacement in any direction (*x*, *y*, or *z*) or 2.5° of angular motion were ruled out. The resulting images were spatially normalized into a standard stereotaxic space using a 12-parameter affine approach and an EPI template image that was resampled to 3 × 3 × 3 mm^3^ voxels. Following this, temporal filtering (0.01 Hz < f < 0.08 Hz) was applied to the time series of each voxel to reduce the effect of low-frequency drifts and high-frequency noise. Any linear trend was then eliminated.

### 2.6. Selection of Region of Interest (ROI)

The regions of interest (ROIs) of insula were defined according to the automated anatomical labeling (AAL) template [19] in the REST tool kit (http://www.resting-fmri.sourceforge.net) [20]. Then, the insula-ROIs were resampled to 3 × 3 × 3 mm^3^ as a mask for the further ReHo analysis.

In order to explore the FC between the insula and the whole brain, we divided the insula into three subregions for both right and left referenced to the previous research [21], including the ventral anterior insula (vAI), dorsal anterior insula (dAI), and posterior insula (PI). The bilateral insula subregions were defined anatomically by drawing insula gray matter on the Montreal Neurological Institute (MNI) 152 standard brain. Each voxel in the insula subregion ROIs (converted to 3 mm resolution) was used as a seed in a whole-brain FC analysis in the DA, NDA, and HC groups.

### 2.7. ReHo Analysis

DPARSF software was used to analyze ReHo maps. Individual ReHo maps were generated by calculating the Kendall coefficient [22] concordance of the time series of a given voxel with those of its nearest neighbors (27 voxels) in a voxel-wise manner. ReHo maps were normalized transformed to standard zReHo maps and then smoothed with a Gaussian kernel of 6 mm (full width at half maximum; FWHM), in order to reduce the effect of individual variations on the Kendall coefficient of concordance value.

### 2.8. FC Analysis

The FC analysis was supported by REST tool kit (http://www.resting-fmri.sourceforge.net) [20]. Global trend, white matter (WM), and cerebrospinal fluid (CSF) were obtained by averaging the time series within the whole brain, WM, and CSF masks, respectively. For each insula-ROI, a seed referenced time course was obtained by averaging the time series of all voxels in the ROI. Then, Pearson's correlation analysis was performed between the seed reference time course and time series of each voxel in the brain in a voxel wise way. And a Fisher's *z*-transform was applied to improve the normality of the correlation coefficients [23]. Six head motion parameters and the mean time series of global signals, WM signals, and CSF signals were introduced as covariates into a random effects model to remove possible effects of head motion, global signal, WM signal, and CSF signals on the results.

### 2.9. Statistical Analysis

Predictive Analytics Software (PASW) Statistics 18 package was employed (IBM Corporation, Armonk, NY, USA) to complete the analyses. Age, education, and HDRS-17 scores were performed by one-way analysis of variance (ANOVA). Gender was compared by means of the chi-square test. Duration of illness and ACT scores were analyzed by independent samples *t*-test. *P* values less than 0.05 were considered to indicate statistical significance.

Insula zReHo values and its subregions FC comparisons were also processed with REST software. Statistical tests across groups were performed using a voxel-based, one-way analysis of covariance (ANCOVA), with age, gender, and education level as covariates. We used AlphaSim correction based on the Monte Carlo simulation algorithm to correct for multiple comparisons, using the following parameters for zReHo: single voxel *P* value = 0.01, FWHM = 6 mm, with 61 × 73 × 61 mm^3^ insula mask, which yielded a corrected threshold of *P* < 0.01, and cluster size >375 mm^3^ (https://afni.nimh.nih.gov/pub/dist/doc/manual/AlphaSim.pdf) and the following parameters for FC: single voxel *P* value = 0.01, FWHM = 6 mm, with 61 × 73 × 61 mm^3^ grey matter mask, which yielded a corrected threshold of *P* < 0.01/6, and cluster size >1431 mm^3^. The post hoc independent samples *t*-test of FC was conducted within a mask showing significant differences obtained from the ANCOVA analysis, with AlphaSim corrections (single voxel *P* value = 0.01, FWHM = 6 mm, which yielded a corrected threshold of *P* < 0.01, and cluster size >216 mm^3^/135 mm^3^ for the left and right vAI, resp.).

Brain regions which exhibited difference among the three groups were further selected as ROIs. Mean FC values were extracted within each of these ROIs for further receiver operating characteristic (ROC) curve analyses. Furthermore, Pearson correlation coefficients were computed between the extracted insula subregions FC values within these ROIs and the clinical assessments of DA patients by PASW 18.0, and the significance level was set at *P* < 0.05 (two tailed).

## 3. Results

### 3.1. Demographic and Clinical Data

As shown in Table 1, DA patients showed significantly lower scores in ACT (*P* < 0.01) compared with NDA patients. There were no significant differences in the age, gender, education, and durations between the groups.

### 3.2. Insula ReHo Results

In the current study, significant differences of zReHo values in the insula between DA, NDA, and HCs were not found.

### 3.3. Insula Subregions FC Results

**Figure 1:**
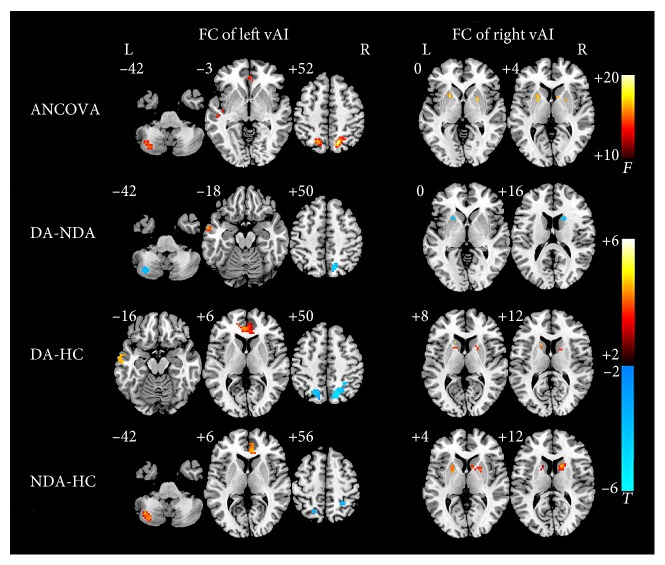
Statistical maps showing vAI FC differences in different brain regions between the DA, NDA, and HC groups. ANCOVA significantly increased in vAI FC among the DA, NDA, and HCs groups (*P* < 0.01/6, AlphaSim corrected). The FC between left vAI with left cerebellum posterior lobe, left MTG, bilateral ACC, and bilateral parietal lobe were increased; the FC between right vAI with the left putamen and right caudate were also increased; the red color bar indicates the *F* value from ANCOVA among the three groups. DA-NDA significantly altered in vAI FC of DA patients compared with that of NDA patients (*P* < 0.01, AlphaSim corrected). DA patients showed increased left vAI FC with MTG, decreased left vAI FC with left cerebellum posterior lobe and right parietal lobe, and decreased right vAI FC with the left putamen and right caudate. DA-HC significantly changes in vAI FC of DA patients compared with that of HCs (*P* < 0.01, AlphaSim corrected). DA patients showed increased left vAI FC with left MTG and bilateral ACC, decreased left vAI FC with bilateral parietal lobe, and increased right vAI FC with the left putamen and right caudate. NDA-HC significantly altered in vAI FC of NDA patients compared with HCs (*P* < 0.01, AlphaSim corrected). The NDA patients showed increased left vAI FC with left cerebellum posterior lobe and right ACC, decreased left vAI FC with bilateral parietal lobe, and increased right vAI FC with the left putamen and right caudate. The color bar indicates the *t* value from independent samples *t*-test between the three groups. ANCOVA, analysis of covariance; DA, depressed asthma; NDA, nondepressed asthma; HCs, healthy controls; FC, functional connectivity; vAI, ventral anterior insula; MTG, middle temporal gyrus.

In the left insula, vAI showed significant altered whole-brain connections among the DA, NDA, and HC groups (see Table 2 and Figure 1). Compared with NDA, DA patients showed decreased connectivity between left vAI and the left cerebellum posterior lobe and right parietal lobe, respectively, and increased connectivity between left vAI and the left middle temporal gyrus (MTG). In addition, compared with HCs, DA patients exhibited increased left vAI FC and both left MTG and bilateral anterior cingulate cortex (ACC) and decreased left vAI FC and the bilateral parietal lobe. Compared with HCs, increased left vAI FC with the left cerebellum posterior lobe and right ACC and decreased left vAI FC with the bilateral parietal lobe were found in NDA patients.

In terms of the right insula (see Table 2 and Figure 1), decreased FC between right vAI and both left putamen and right caudate were found in the DA patients compared with that in the NDA patients. Compared with HC, DA, and NDA patients, both showed increased right vAI FC with the left putamen and right caudate.

### 3.4. Correlations between FC and Scales

The present study used partial correlation analysis to explore the relationships between mean FC values in ROIs (brain regions showed differences among the three groups by ANCOVA) and clinical assessments. No significant correlations were found between FC values in ROIs and HDRS-17, ACT scores, respectively, either in DA or in NDA group.

### 3.5. ROC Analyses

**Figure 2:**
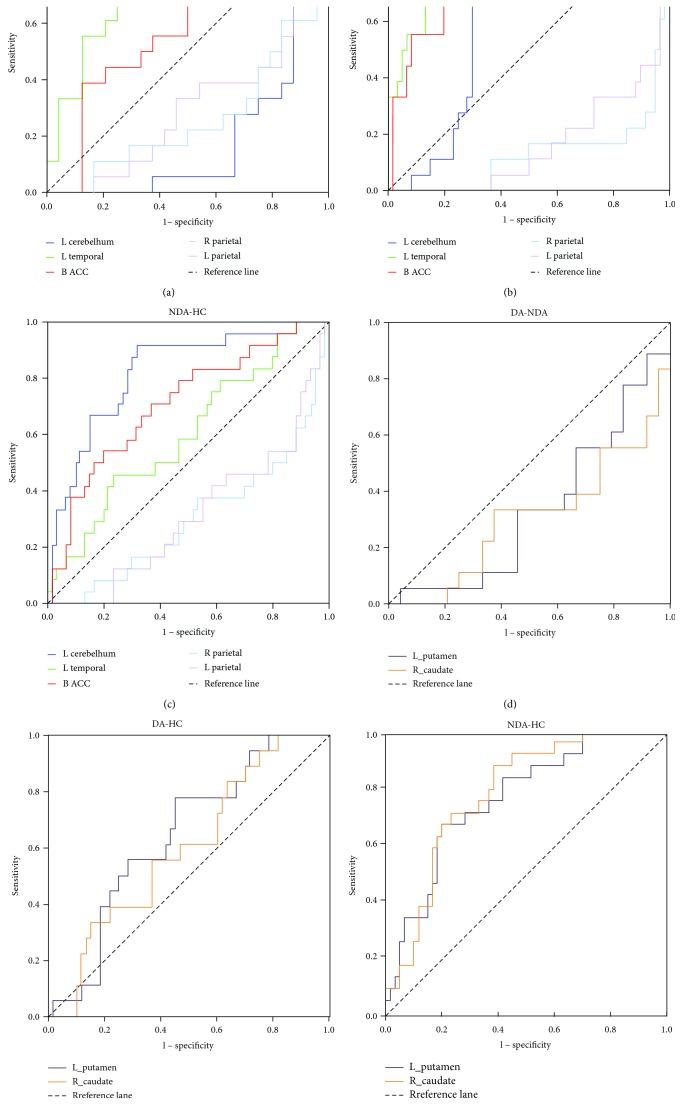
ROC analyses. (a) ROC analyses differentiate DA from NDA patients by using FC values between left vAI with left cerebellum posterior lobe, left MTG, bilateral ACC, right parietal lobe, and left parietal lobe. The areas under the ROC curve for FC between left vAI with left cerebellum posterior lobe, left MTG, bilateral ACC, right parietal lobe, and left parietal lobe were 0.194 (*P* < 0.001; 95% CI: 0.058–0.331), 0.759 (*P* < 0.01; 95% CI: 0.060–0.912), 0.632 (*P* > 0.5; 95% CI: 0.450–0.796), 0.257 (*P* < 0.01; 95% CI: 0.102–0.412), and 0.294 (*P* < 0.05; 95% CI: 0.134–0.454), respectively. (b) ROC analyses differentiate DA from HC by using FC values between left vAI with left cerebellum posterior lobe, left MTG, bilateral ACC, right parietal lobe, and left parietal lobe. The areas under the ROC curve for FC between left vAI with left cerebellum posterior lobe, left MTG, bilateral ACC, right parietal lobe, and left parietal lobe were 0.700 (*P* < 0.01; 95% CI: 0.588–0.812), 0.831 (*P* < 0.001; 95% CI: 0.709–0.952), 0.828 (*P* < 0.001; 95% CI: 0.721–0.935), 0.129 (*P* < 0.001; 95% CI: 0.024–0.233), and 0.159 (*P* < 0.001; 95% CI: 0.057–0.262), respectively. (c) ROC analyses differentiate NDA from HC by using FC values between left vAI with left cerebellum posterior lobe, left MTG, bilateral ACC, right parietal lobe, and left parietal lobe. The areas under the ROC curve for FC between left vAI with left cerebellum posterior lobe, left MTG, bilateral ACC, right parietal lobe, and left parietal lobe were 0.820 (*P* < 0.001; 95% CI: 0.720–0.920), 0.599 (*P* > 0.5; 95% CI: 0.465–0.733), 0.709 (*P* < 0.01; 95% CI: 0.586–0.833), 0.301 (*P* < 0.01; 95% CI: 0.173–0.429), and 0.314 (*P* < 0.01; 95% CI: 0.189–0.438), respectively. (d) ROC analyses differentiate DA from NDA patients by using FC values between right vAI with the left putamen and right caudate. The areas under the ROC curve for FC between left vAI with the left putamen and right caudate were 0.336 (*P* > 0.05; 95% CI: 0.1700.502) and 0.306 (*P* < 0.05; 95% CI: 0.141–0.470), respectively. (e) ROC analyses differentiate DA patients from HC by using FC values between right vAI with the left putamen and right caudate. The areas under the ROC curve for FC between left vAI with the left putamen and right caudate were 0.644 (*P* > 0.05; 95% CI: 0.510–0.777) and 0.599 (*P* > 0.05; 95% CI: 0.459–0.739), respectively. (f) ROC analyses differentiate NDA patients from HC by using FC values between right vAI with the left putamen and right caudate. The areas under the ROC curve for FC between left vAI with left putamen and right caudate were 0.760 (*P* < 0.001; 95% CI: 0.651–0.868) and 0.778 (*P* < 0.001; 95% CI: 0.677–0.880), respectively. DA, depressed asthma; NDA, nondepressed asthma; HC, healthy controls; ACC, anterior cingulate cortex; FC, functional connectivity; vAI, ventral anterior insula; MTG, middle temporal gyrus.

The mean FC values between left vAI and left cerebellum posterior lobe, left MTG, bilateral ACC, and bilateral parietal lobe were extracted, respectively, for the further ROC analyses (see Table 3 and Figure 2). The area under the curve (AUC) in FC between left vAI and left cerebellum posterior lobe was 0.7 (*P* < 0.01) which distinguished DA from HCs preferably (Table 3, Figure 2(b)), and it also differentiated NDA from HCs with an AUC of 0.82 (*P* < 0.001) (Table 3, Figure 2(c)). Similarly, FC between left vAI and bilateral ACC also significantly distinguished HCs from the DA and NDA groups (Table 3, Figures 2(b) and 2(c)). And the FC between left vAI and left MTG significantly distinguished DA from the NDA and HC groups (Table 3, Figures 2(a) and 2(b)). In addition, both the FC between right vAI and both the left putamen and the right caudate only significantly differentiated NDA from HCs (Table 3, Figure 2(f)), without distinguishing DA from NDA or DA from HCs (Table 3, Figures 2(d) and 2(e)).

## 4. Discussions

In the present study, we employed the method of ReHo to measure the spontaneous activity of insula, as well as to investigate the relationship between insula subregions FC with whole brain in DA, NDA, and HCs. The results demonstrated that compared with HCs, both DA and NDA patients have no significant differences in spontaneous activity in the insula. However, to the best our knowledge, we demonstrated for the first time that asthmatic patients displayed altered insula FC compared with HCs. The present study found that DA patients showed increased FC between left vAI and left MTG compared with both the NDA and HC groups, and it could separate DA patients from NDA and HCs. In addition, when compared with HCs, the DA and NDA patients both exhibited increased FC between left vAI and right ACC, decreased FC between left vAI and bilateral parietal lobe, increased FC between right vAI and left putamen, and increased FC between right vAI and right caudate. Furthermore, the increased FC between left vAI and right ACC could differentiate HCs from both the DA and NDA patients. And the increased FC between right vAI and both the left putamen and the right caudate could separate NDA patients from HCs.

In the current study, compared with HCs, both the DA and NDA patients did not show significant differences of ReHo in the insula, which was consistent with the finding in our previous study that asthmatic patients did not exhibit abnormal ReHo in the insula [24]. Many studies indicated that the insula is associated with the perception of dyspnea both in patients with respiratory diseases [11, 25, 26] and healthy subjects [13], because dyspnea has a sensory and an effective dimension [27]. However, Peiffer et al. [28] explored dyspnea-related brain activation in healthy subjects, and the altered activity in the insula was not found during dyspnea. Although the anterior insula is a critical brain region involved in the experience of negative emotions [29], many fMRI research of depression did not report abnormal activity in the insula [30, 31]. These findings further supported our study that significant differences of ReHo in the insula were not found between the DA and NDA patients. Thus, the relationship between spontaneous activity of the insula with asthma-specific symptoms and emotions needs further study.

A functional imaging study indicated that MTG belongs to the visual recognition circuit, which is assumed to play an important role in processing facial stimuli [32]. Further, it also regulates semantic processing, the processing of emotional information and cognitive regulation [33]. Cao et al. [33] reported that increased spontaneous brain activity in the left MTG may cause emotional dysregulation, thus increases the vulnerability to impulsive and suicidal behavior in major depression disorder. Increased gray matter volume in the MTG was also found in the late onset depression, which suggested that it would be an anatomical basis for emotional dysregulation and impaired decision making [34]. In addition, neuroimaging revealed that vAI is connected to regions representing sensory inputs associated with affective experience [21, 35]. In the current study, ROC analyses demonstrated that the increased FC between left vAI and left MTG as an independent variable performed well in differentiating DA from both the NDA and HC groups. The previous study also reported decreased neural activity in MTG in patients with depression disorder [36]; however, the similar finding was not found in DA patients. Therefore, the increased FC between left vAI and left MTG in DA patients would be associated with emotional dysregulation.

Rosenkranz and Davidson [10] demonstrated that the anatomical projections of ACC and insula implicate these structures in monitoring changes in physiological status, integrating this information with external sensory, cognitive, and emotional information and directing the appropriate behavioral and peripheral physiological responses. ACC and insula may be hyperresponsive to asthma-specific emotional and afferent physiological signals, which may contribute to the dysregulation of peripheral processes [11]. In the current study, both DA and NDA patients showed increased FC between left vAI and ACC compared with HCs. It was consistent with the findings of von Leupoldt and Dahme [37, 38] that patients with dyspnea show increased activity in the ACC. Furthermore, greater activity in the perigenual ACC seems to reflect greater reactivity and is associated with greater airway inflammation, a more robust alpha amylase response, and a greater stress-induced increased in proinflammatory cytokine mRNA expression in airway cells [39]. Thus, the increased FC between left vAI and ACC in asthmatic patients would be possible to be associated with asthma-specific inflammation and emotions.

The parietal lobe is primarily responsible for the integration of sensory information, both tactile and perceived, as well as spatial recognition and processing of both language and memory [40]. Zhang et al. [41] explored the brain activity in healthy subjects that experienced experimentally induced low back pain and found that the right inferior parietal lobe of these subjects showed a decreased spontaneous activity. Moreover, they suggested that these changes may account for the recognition, execution, and emotional and memory process involved in acute pain [41]. In patients with headache, the gray matter density in the bilateral parietal lobe was also decreased [42]. In the present study, asthmatic patients showed decreased FC between left vAI and bilateral parietal lobe, which was similar to above findings. The neural structures that promote dyspnea and pain are shared [27], we deduced that the decreased FC between left vAI and bilateral parietal lobe would be the underpinning of cognitive in asthma.

The putamen and caudate are believed to contribute to sensorimotor activity and cognition, respectively [43]. An fMRI study of the normal fear response in healthy subjects revealed increased putamen activation in response to a fearful situation [44]. In patients with panic disorder, the putamen also showed abnormal function indicating that the subcortical mediated panic-related fight or flight response may be abnormal [45]. In addition, the putamen also plays a critical role in gating respiratory information to the cortex [46]. Since vAI is connected with visceromotor regions [21, 35], thus, we speculated that the increased FC between right vAI and left putamen in asthmatic patients may involve in the asthma-specific fear. Furthermore, in the present study, this increased FC differentiated NDA patients from HCs. It further supported our speculations.

Previous studies reported that caudate nucleus has stronger anatomical links with the prefrontal cortex, and caudate is demonstrated to be involved in more cognitive tasks [47, 48]. For example, Quevedo et al. [49] found patients with depression showed heightened caudate and insula to ventral striatum connectivity, which suggested that these patients may intend to have more behavioral planning and goal-oriented cognitions for negative outcomes. Therefore, the increased FC between right vAI and right caudate in the current study was possibly associated with the hyperattention of asthma symptoms.

On the basis of previous studies, these abnormal vAI FC are involved in asthma-specific symptoms including inflammation, cognition, fear, hyperattention, and emotion. Whether these abnormal FC could be used as biomarkers to predict depression in asthma needs to be further demonstrated with large samples in the future study.

There were some limitations in the current study. First, it was a nonrandomized study with a relatively small sample size. Since all asthmatic patients received the same kind of pharmacotherapy, the effect of antiasthma drugs on brain functions needs further exploration. Second, the generalizability of the results might have been reduced due to the sampling strategy. Third, we just assessed the depression severity and asthma control level in the present study; more cognitive-related tests were required adequately to describe the patients' cognitive profile. To overcome these limitations, studies with larger sample sizes are needed in the future.

## 5. Conclusions

The current study showed for the first time the evidence of altered vAI FC of depression that would be involved in the neuropathology of depression in asthma.

## Figures and Tables

**Table 1:** Demographics and clinical characteristics of participants.

	DA (*n* = 18)	NDA (*n* = 24)	HCs (*n* = 60)	*P* value
Age (years)	53.61 ± 9.08^∗^	50.58 ± 10.57	45.78 ± 14.49	0.051^a^
Gender (male/female)	9/9	9/15	24/36	0.688^b^
Education (years)	11.89 ± 2.56	11.75 ± 2.64	12.42 ± 3.57	0.639^a^
Duration of asthma (years)	22.86 ± 20.19	21.42 ± 19.27	—	0.815^c^
HDRS-17 scores	11.06 ± 4.40^∗∗^^††^	2.21 ± 1.47^#^	0.93 ± 1.34	<0.001^a^
ACT scores	15.00 ± 4.38	19.58 ± 4.31	—	0.002^c^

Note: data are expressed as mean ± standard deviation. ^a^One-way ANOVA; ^b^Chi-square test; ^c^Independent-sample *t*-test. DA versus HC, ^∗^*P* < 0.05, ^∗∗^*P* < 0.001; DA versus NDA, ^††^*P* < 0.001; NDA versus HC, ^#^*P* < 0.05. DA: depressed asthma; NDA: nondepressed asthma; HCs: healthy controls; HDRS-17: 17-item Hamilton Depression Rating Scale; ACT: asthma control test.

**Table 2:** The FC between vAI with the whole brain among DA, NDA, and HCs.

Peak area	BA	Side	MNI coordinates			Voxels	Peak *t*-value
			X	Y	Z		
*FC between left vAI and following brain regions*							
*ANCOVA*							
Cerebellum posterior lobe	—	L	−39	−69	−39	66	12.857
Middle temporal gyrus	21	L	−51	−21	−6	77	10.0207
ACC	32	B	3	42	0	107	10.4837
Parietal lobe	7	R	15	−66	51	161	20.4306
Parietal lobe	7	L	−18	−63	54	79	13.9602
*DA-NDA*							
Cerebellum posterior lobe	—	L	−36	−72	−42	23	−3.6785
Middle temporal gyrus	21	L	−66	−12	−6	28	4.4333
Parietal lobe	7	R	15	−66	51	52	−4.8216
*DA-HCs*							
Middle temporal gyrus	20	L	−39	−12	−6	77	4.4481
ACC	32	B	−3	39	3	107	4.5744
Parietal lobe	7	R	15	−66	51	161	−6.2223
Parietal lobe	7	L	−18	−63	54	79	−5.2783
*NDA-HCs*							
Cerebellum posterior lobe	—	L	−39	−69	−39	66	4.7411
ACC	32	R	6	36	6	62	3.862
Parietal lobe	7	L	−18	−63	48	20	−3.2677
Parietal lobe	7	R	30	−45	60	24	−3.6245

*FC between right vAI and following brain regions*							
*ANCOVA*							
Putamen	—	L	−21	9	0	54	10.0985
Caudate	—	R	15	12	15	82	13.983
*DA-NDA*							
Putamen	—	L	−21	15	0	7	−3.2276
Caudate	—	R	15	12	18	12	−3.55
*DA-HCs*							
Putamen	—	L	−15	18	9	7	4.0056
Caudate	—	R	21	6	9	6	2.8772
*NDA-HCs*							
Putamen	—	L	−21	9	0	52	4.6445
Caudate	—	R	15	12	15	82	4.985

Note: ANCOVA threshold was set at *P* < 0.01/6 (AlphaSim-corrected, cluster size >1431 mm^3^). The independent *t*-test threshold was set at *P* < 0.01 (AlphaSim-corrected, cluster size >216 mm^3^ for left vAI and 135 mm^3^ for right vAI). X, Y, Z: coordinates of primary peak locations in the MNI space; MNI: Montreal Neurological Institute space; BA: Brodmann area; vAI: ventral anterior insula; L: left; R: right; B: bilateral; DA: depressed asthma; NDA: nondepressed asthma; HCs: healthy controls; ANCOVA: one-way analysis of covariance.

**Table 3:** ROC analyses for separating different groups.

Brain regions	AUC	*P* value	95% CI	Sensitivity	Specificity	Cut-off point
*FC between the left vAI and the following brain regions*						
*Left cerebellum posterior lobe*						
DA-NDA	0.194	0.001	0.058–0.331	0.056	0.333	−0.0095^a^
DA-HCs	0.700	0.010	0.588–0.812	0.944	0.583	−0.1290
NDA-HCs	0.820	<0.001	0.720–0.920	0.917	0.683	−0.0808
*Left middle temporal gyrus*						
DA-NDA	0.759	0.004	0.606–0.912	0.778	0.708	0.0932
DA-HCs	0.831	<0.001	0.709–0.952	0.778	0.833	0.0941
NDA-HCs	0.599	0.157	0.465–0.733	0.458	0.767	0.0632
*Bilateral anterior cingulate cortex*						
DA-NDA	0.623	0.178	0.450–0.796	0.833	0.458	0.1389
DA-HCs	0.828	<0.001	0.721–0.935	0.833	0.783	0.1450
NDA-HCs	0.709	0.003	0.586–0.833	0.542	0.800	0.1554
*Right parietal lobe*						
DA-NDA	0.257	0.008	0.102–0.412	0.278	0.292	−0.1453
DA-HCs	0.129	<0.001	0.024–0.233	0.222	0.083	−0.1336
NDA-HCs	0.301	0.005	0.173–0.429	0.500	0.150	−0.1057
*Left parietal lobe*						
DA-NDA	0.294	0.024	0.134–0.454	0.444	0.167	−0.2058
DA-HCs	0.159	<0.001	0.057–0.262	0.333	0.117	−0.1446
NDA-HCs	0.314	0.008	0.189–0.438	0.542	0.117	−0.1489

*FC between right vAI and following brain regions*						
*Left putamen*						
DA-NDA	0.336	0.071	0.170–0.502	0.111	0.542	0.3462
DA-HCs	0.644	0.066	0.510–0.777	0.778	0.550	0.2277
NDA-HCs	0.760	<0.001	0.651–0.868	0.667	0.800	0.3013
*Right caudate*						
DA-NDA	0.306	0.033	0.141–0.470	0.389	0.250	0.2506
DA-HCs	0.599	0.204	0.459–0.739	0.833	0.367	0.1431
NDA-HCs	0.778	<0.001	0.677–0.880	0.875	0.617	0.2381

Note: ^a^This cut-off point resulted in a sensitivity of 5.6% and a specificity of 33.3% while DA patients separating from NDA patients. The means of other cut-off points were similar. ROC: receiver operating characteristic; AUC: area under the curve; CI: confidence interval; DA: depressed asthma; NDA: nondepressed asthma; HCs: healthy controls.
